# World Health Organization AWaRe framework for antibiotic stewardship: Where are we now and where do we need to go? An expert viewpoint

**DOI:** 10.1017/ash.2023.164

**Published:** 2023-04-26

**Authors:** Steward Mudenda, Victor Daka, Scott K. Matafwali

**Affiliations:** 1 Department of Pharmacy, School of Health Sciences, University of Zambia, Lusaka, Zambia; 2 Department of Public Health, Michael Chilufya Sata School of Medicine, Copperbelt University, Ndola, Zambia; 3 Clinical Research Department, Faculty of Infectious and Tropical Diseases, London School of Hygiene & Tropical Medicine, London, United Kingdom

## Abstract

The AWaRe classification categorizes antibiotics and is a tool for antimicrobial stewardship. To combat antimicrobial resistance, prescribers must adhere to the AWaRe framework, which promotes the rational use of antibiotics. Therefore, increasing political will, dedicating resources, building capacity, and improving awareness and sensitization campaigns may promote adherence to the framework.

Antimicrobial resistance (AMR) is a significant global health threat to humans, animals, agriculture, and the environment.^1,2^ Addressing AMR requires the establishment and implementation of antimicrobial stewardship (AMS) programs.^3^

In 2017, the World Health Organization (WHO) developed the Access, Watch, and Reserve (AWaRe) classification system of antibiotics as part of AMS.^4^ This was a great milestone in the fight against AMR because a more objective, user-friendly tool became available to organize the antibiotics. The WHO AWaRe framework categorizes antibiotics according to their spectrum of activity and potential to develop resistance.^4^ The Access group contains antibiotics used in the first- and second-line treatment of infections.^5^ The Watch group contains broad-spectrum antibiotics with a higher potential of developing resistance.^5^ The Reserve group contains last-resort antibiotics used for multidrug-resistant infections.^5^ Furthermore, the framework promotes the responsible use of antibiotics by classifying them according to their importance in human medicine and promoting their appropriate use. The framework also includes key components such as prescribing guidelines and surveillance systems to monitor antimicrobial use (AMU) and AMR, as well as promoting research and development of new antibiotics and alternative treatment options.^4,5^ By 2023, the WHO AWaRe tool recommends that 60% of all prescribed antibiotics must belong to the Access group.^4^ Therefore, developing, implementing, and adhering to the WHO AWaRe framework promotes AMS and reduces the inappropriate use of antibiotics.

## Prior successes of the WHO AWaRe tool

Since its introduction, progress has been made in different regions of the world in implementing the AWaRe framework.^6^ Significant progress has been reported by countries that have adopted national policies and guidelines on AMS and have implemented measures such as prescribing guidelines and surveillance systems to monitor AMU and AMR. Thus, countries that implemented the AWaRe framework are fully guided on the antibiotic to use for specific diseases. Additionally, prescribers are guided on the dosage of antibiotics for specific infections. This guidance has improved adherence to the recommended antibiotic prescribing guidelines. From the Zambian point of view, implementation of the AWaRe tool is still in its infancy, but hospitals where prescribers adhered to this AMS strategy reported low AMR challenges.^7^ Therefore, the political will demonstrated by the Government of the Republic of Zambia to support the implementation of this AMS strategy will further prevent and reduce AMR cases.

## Challenges faced in implementing the WHO AWaRe framework of antibiotics

The challenges reported here were from various countries but also apply to Zambia. One major challenge has been lack of awareness and education about appropriate antibiotic use. Many healthcare providers and patients are still not fully aware of the risks associated with the nonprudent AMU and the consequences of AMR. This lack of awareness is one of the reasons why AMR continues to be a serious global public health concern. Additionally, the lack of awareness of the WHO AWaRe framework of antibiotics influences healthcare workers to prescribe and dispense antibiotics without following the guidelines, worsening the AMR challenge.^5,8^ Another major challenge is limited resources and capacity to implement the framework in many countries, especially in lower- and middle-income countries.^9^ Additionally, the low availability of affordable and good-quality antibiotics affects the sustainable implementation of the AWaRe system.^9^ A lack of political will in many countries continues to be a barrier to the implementation of the AWaRe framework of antibiotics. Therefore, these challenges may affect the strengthening of AMS, surveillance and monitoring systems of AMU and AMR, including the research and development of new antibiotics and alternative treatment options.

## Recommendations for improvement of using the WHO AWaRe tool

To tackle these challenges and further the progress in the future, we recommend the following measures:

- To push for further progress, continued collaborative efforts are needed to improve AMS. These efforts include increasing awareness and education about appropriate antibiotic use, strengthening surveillance and monitoring systems, and promoting research and development of new antibiotics and alternative treatment options. Healthcare authorities must increase awareness and sensitization campaigns and educational activities that may promote the use of and adherence to the WHO AWaRe classification of antibiotics. Healthcare workers, such as physicians, pharmacists, nurses, dental surgeons, and clinical officers, must be educated about the framework because they are the main prescribers and dispensers of antibiotics both in public and private healthcare facilities.
- Additionally, it is important to explore the potential role of global collaborations and partnerships in promoting AMS. Implementing and sustaining the WHO AWaRe classification of antibiotics may require additional resources such as human and funding needs. Thus, we urge countries to collaborate with other countries and organizations and learn from other nations that have successfully implemented the AWaRe framework of antibiotics.
- It is also important to address the social determinants of health, such as poverty, education, and access to healthcare, in addressing AMR and promoting the prudent use of antibiotics. This attention to social issues may help reduce the burden of infections and noncommunicable diseases, leading to a reduction in the use of antibiotics.
- Furthermore, the One Health approach should be promoted when tackling AMR as guided by the Global Action Plan (GAP) on AMR. This approach ensures that AMR is addressed across all sectors, including human, animal, agriculture, the environment and all its ecological systems. Therefore, professionals from the human, animal, agriculture, and environmental sectors must collaborate for the common good of tackling AMR. Furthermore, management of infections across sectors should be conducted according to the WHO AWaRe framework of antibiotics.
- AMS programs need to be extended from centralized, larger healthcare facilities to smaller healthcare facilities. With many patients seen in smaller healthcare facilities like clinics and first-level hospitals, the risks of misusing and overusing antibiotics are high and require the adoption of the AWaRe framework of antibiotics in such settings. A key component in the development and sustainability of these programs needs a strong research and development framework that will respond to the needs of individual countries. This will ensure that the programs evolve and are current.
- Political will from governments is critical in establishing, implementing, and sustaining healthcare programs such as AMS. The need for healthcare workers to adhere to the WHO AWaRe framework of antibiotics requires political will because policies are developed and implemented by authorities. Successful implementation of the AWaRe framework of antibiotics may require many political players, such as those in leadership politics, bureaucratic politics, interest group politics, beneficiary politics, budget politics, and external actor politics.

## Experiences in Zambia on the use of the AWaRe classification of antibiotics

In Zambia, there are gaps and lack of knowledge among healthcare workers on the use the AWaRe tool because studies have reported high prescribing of Watch antibiotics.^7,8,10^ In all studies, ceftriaxone, a Watch antibiotic, was being overused (Table 1). This contrasts with the WHO recommendations. As the country heightens its AMS activities in hospitals and other sectors, AMU will likely improve.

**Table 1.** The Most Prescribed Antibiotic in Zambian Hospitals Between 2020 and 2022

Authors	Most Prescribed Antibiotic	AWaRe Category
Mudenda et al, 2022^10^	Ceftriaxone	Watch
Kalungia et al, 2022^7^	Ceftriaxone	Watch
Mudenda et al, 2023^8^	Ceftriaxone	Watch

In conclusion, the WHO AWaRe classification of antibiotics provides a valuable framework for monitoring AMU and addressing AMR. Nevertheless, there is still much work to be done to ensure the widespread implementation of this classification framework and its effectiveness in reducing AMU and AMR. By addressing the challenges and adopting a multifaceted approach, we can make progress in the fight against AMR.
